# *SALT*: transfer learning-based threat model for attack detection in smart home

**DOI:** 10.1038/s41598-022-16261-9

**Published:** 2022-07-18

**Authors:** Pooja Anand, Yashwant Singh, Harvinder Singh, Mohammad Dahman Alshehri, Sudeep Tanwar

**Affiliations:** 1 Department of Computer Science and Information Technology, Central University of Jammu, Rahya Suchani, Jammu and Kashmir 181143 India; 2 Leaders Institute, Woolloongabba, Brisbane, QLD -4102 Australia; 3 Centre for Artificial Intelligence Research and Optimisation, Torrens University Australia, Fortitude Valley, QLD 4006 Australia; 4 Department of Computer Science, College of Computers and Information Technology, Taif University, P.O. Box 11099, Taif, 21944 Saudi Arabia; 5 Department of Computer Science and Engineering, Institute of Technology, Nirma University, Ahmedabad, Gujarat 382481 India

**Keywords:** Electrical and electronic engineering, Computer science

## Abstract

The next whooping revolution after the Internet is its scion, the Internet of Things (IoT), which has facilitated every entity the power to connect to the web. However, this magnifying depth of the digital pool oil the wheels for the attackers to penetrate. Thus, these threats and attacks have become a prime concern among researchers. With promising features, Machine Learning (ML) has been the solution throughout to detect these threats. But, the general ML-based solutions have been declining with the practical implementation to detect unknown threats due to changes in domains, different distributions, long training time, and lack of labelled data. To tackle the aforementioned issues, Transfer Learning (TL) has emerged as a viable solution. Motivated by the facts, this article aims to leverage TL-based strategies to get better the learning classifiers to detect known and unknown threats targeting IoT systems. TL transfers the knowledge attained while learning a task to expedite the learning of new similar tasks/problems. This article proposes a learning-based threat model for attack detection in the Smart Home environment (SALT). It uses the knowledge of known threats in the source domain (labelled data) to detect the unknown threats in the target domain (unlabelled data). The proposed scheme addresses the workable differences in feature space distribution or the ratio of attack instances to a normal one, or both. The proposed threat model would show the implying competence of ML with the TL scheme to improve the robustness of learning classifiers besides the threat variants to detect known and unknown threats. The performance analysis shows that traditional schemes underperform for unknown threat variants with accuracy dropping to 39% and recall to 56.

## Introduction

Internet of Things (IoT) is getting matured by employing an umbrella of technologies working together to connect smart devices to the Internet. With such expansion, there is a need to have ubiquitous control over these smart devices^1^. Such as our things, fridges, homes, TVs, cars, cameras, transportation, medicals, surgeries, cities, grids, agriculture, industries, and defense. Moreover, IoT is a blend of diverse technologies working together in an IoT communication stack to bestow the preeminent services in diversified application areas. According to the predicting agency, the number of smart devices will increase up to 41 billion by 2027^2^. Such a massive increase in the usage of smart devices will end up generating the data up to Geopbytes (1030) even. Moreover, embracing such a technological shift in our lives has intensified the risk of cyber-attacks and privacy breaches. For example, the Palo Alto Threat report under, Unit 42 substantiates the declination of IoT with supporting numbers for growing attacks^3^. The study claims the unencrypted IoT data traffic is ruling with 98% and 83% medical devices running the antiquated operating systems. Additionally, depicting the smart security cameras ushering with 33% security concerns, and the smart printers ruling out with 24%^4^. In relation to growing cyber-attacks in IoT, that too has turned to billions following the IoT with roundabout a three-fold accretion in attack traffic^5,6^.

These statistics have been ruled out in the IoT realm, as these systems are integrated with resource constraint nodes and generally located in unattended environments. Thus, deploying traditional security mechanisms to maintain privacy, access control, integrity, and authentication in smart systems becomes a challenging task^7^. Moreover, these smart devices become part of countless smart services with inherent vulnerabilities as an easy entry point to penetrate. And the negligent manufacturers and unaware users of these smart devices aid in easy playing. The successful launch of several IoT-based botnet attacks also exhilarates its feeble state by shutting down the system with denial of service (DoS)^8^. Even the hackers earn well with cyber assaults collecting good ransom with ransomware attacks. With its many more variants, cryptocurrency could be mined, and privacy could be hindered. All these factors looming on the IoT horizon pose a pronounced risk to the sustainability of IoT in this digital era. The society being served with the vital services nurtured by IoT necessitates the accelerated development of robust, resilient security mechanisms^9^.

Several signature-based and learning-based mechanisms exist in the literature to shield the IoT system. Moreover, threat vectors and attacks are also evolving rapidly, making them inefficient to the growing needs of an IoT system. Thus, opening the ways for intelligent detection engines to adopt the paradigm of transferring knowledge as humans do, to learn with the changing attack pattern and behavior, employed under transfer learning^10^. Even though the learning models are the germane tools for discerning the ‘regular’ and ‘irregular’ behavior of nodes in an IoT system and the interaction among them to provide vital services^11^. These machine learning (ML) classifiers fail to detect unknown threats, usually the mutations of forgoing known threats. Classifiers generally consider them the same and sentence to detect the unknown threats without any specific mechanism. The study articulating the compounding security implications evidenced in smart systems^12^, demands the shift towards identifying the known and unknown threats. Consequently, in this research work, we have proposed a TL-based threat model with baseline ML classifiers to detect the known and unknown threats in the smart home environment.

State-of-the-art threat modelling methods like STRIDE (Spoofing, Tampering, Repudiation, Information Disclosure, DoS, and Elevation of Privilege), DREAD (Damage, Reproducibility, Exploitability, Affected Users, and Discoverability), etc., have been the generalized schemes, initially developed for IT systems and not cover the application of intelligent trends for threat detection. Although, the identification of different known threats under the specific categorization has been generalized. But unknown threats have never been part of those threat models. The studied literature evidence that the tools like Microsoft threat modelling tools are used to develop threat models based on the basic STRIDE approach for the smart environment. However, as per the authors’ knowledge, no specific smart threat modelling approach has been proposed to cover both known and unknown threats in smart systems. For example, authors in^13^ present an application of TL to understand well variants of threat insinuations detecting over the general learning methods. In continuation, Sameera et al.^14^ detected unknown threats by exploiting manifold alignment in the deep transductive TL model. Later, Zhang et al.^15^ proposed a domain adversarial NN-based TL approach to detect network attacks in smart grid.

**Figure 1:**
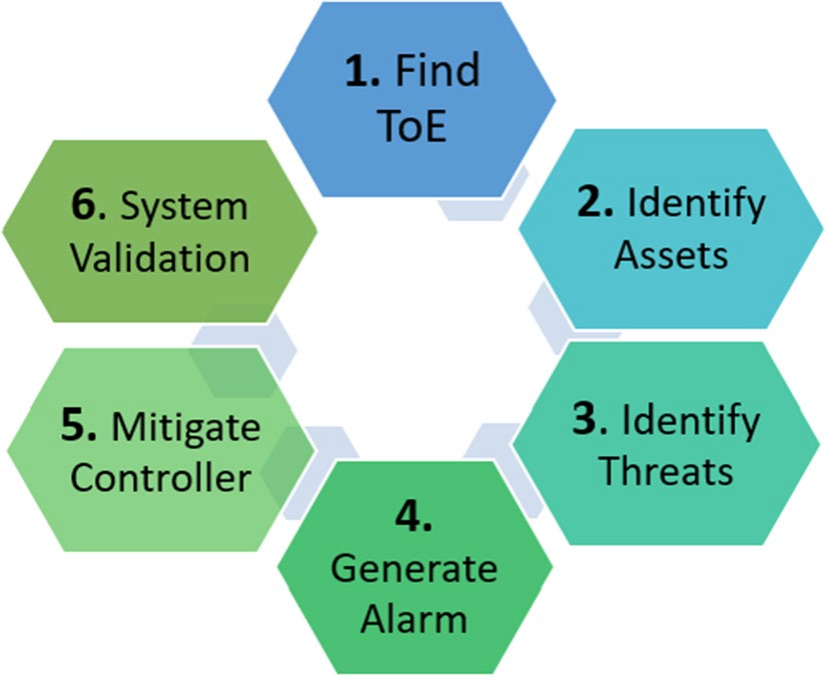
Stages of threat modelling process.

Motivated by the aforementioned discussion, in this paper, we have proposed the TL-based smart threat modelling approach to detect the known and unknown threats in the smart home environment by analyzing the network traffic and leveraging the knowledge acquired while detecting the known threats. Figure 1 shows the various stages of the proposed threat modelling process, which are further elaborated in “The proposed threat modelling methodology” section. In SALT, the targeted smart application/device with identified assets is taken as input by the intelligent detection engine for further system analysis. For known threats, the collected data of the smart device undergoes preprocessing, followed by preprocessed feeds to learning models to detect the system status of being under threat or not. Specifically, for unknown threats, a domain adaptation-based TL scheme is chosen, which facilitates the transfer of knowledge from a domain containing adequate training data, i.e., the source domain, to a similar target domain having very less or no labelled training data at all.

The literature evidence that the growing depth of the digital pool with innumerable IoT devices has made the infant smart world more susceptible to penetrations and cyber-attacks. The key motivation for this study to leverage the gap in the literature are listed below:

- The importance of smart services, AI-based schemes, and their security is questionable with widening cyber-attack surfaces covering even our homes, which have become smart in the recent years compromising our privacy. The resilient and intelligent threat model will strengthen the security of smart homes.
- The existing literature essentially focused either on modelling the known threats or the common vulnerabilities being part and parcel of smart devices, with less emphasis on TL-based intelligence and unknown threats in threat modelling.
- Thus, there is a need for an AI-entrusted threat model with an emerging TL-based scheme to detect the state of a system for different system dynamics and new threat variants emerging in the same or different environment. Therefore, the competence of threat models for self-adaptation in diverse operating scenarios will be a focal point for their deployment in smart applications in real life.

Authors across the globe have presented many solutions^16–23^ to detect cyber-threats in IoT. Pajouh et al.^16^ employed a two-tier classification approach comprising K-Nearest Neighbor (certainty Factor version) and Naïve Bayes. Gao et al.^17^ designed an extreme learning machine to identify suspicious behaviors in smart set-ups. Later, Kumar et al.^18^ proposed an intelligent decentralized scheme for threat detection, employed on fog nodes. Muder et al.^19^ used the recursive neural networks for threat detection. Khraisat et al.^20^ employed a decision tree (C5 classifier) and support vector machine (One-class). In another recent work, Al-Hawawreh et al.^21^ employed a deep learning-based scheme. But they mainly focused on known threats, whose signatures are either stored in the database or the learning model has learned their behavior. However, unknown threats remain undetected by the learning model and the system collapses. This is because most machine learning algorithms assume that the training data and the testing data have same distribution and they are in the same feature space. This assumption does not hold in most real life scenarios. Thus, problem becomes more serious for smart devices, due to their exponential number, constrained resources, and inherent vulnerabilities. And they become easy targets to be the part of a botnet. From last few years, TL has emerged as a potential ground in areas of image identification and natural language processing, but studies using TL in threat detection are still lacking. Moreover, the applicability of the few discussed works in TL^22,23^ is limited due to the use of structured dataset like NSL-KDD or validating through hypotheses on different threats within the same dataset. The TL-based proposed solution can improve the competence of learning models for self-adaptation to detect the different variants of threats.

The key contributions of this work are as follows.

- We present a threat modelling methodology, working in six well-illustrated phases with data flow diagrams (DFDs) upto level-2, about the smart-home environment to identify whether a smart application/device is under known/unknown threat or not.
- We propose SALT, a TL-based threat model for attack detection in smart homes, to make the proposed model intelligent and adaptable to detect the new evolving variants of threats. This can be achieved by applying the domain adaptation paradigm of the TL scheme with customized classifiers for the problem. Also, the correlation analysis of different threats on network dataset of TON-IoT is given.
- We present an algorithm for the proposed threat model, SALT with ML classifier to detect the system is under known threat / not and TL-based classifier to detect the unknown threats. Finally, the theoretical proof to detect whether the system is under the known or unknown threat is given with the threshold decidability factor. Also, the performance analysis of general ML-based solutions on TON-IoT dataset to detect known and unknown threat variants is given.

The rest of the paper is organized as follows. “State-of-the-art” section provides the related work for this study, covering both known threats and unknown threats with learning. “The proposed threat modelling methodology” section discusses the threat modelling methodology with DFDs and the proposed intelligent threat model; SALT is detailed in “SALT: transfer learning-based threat model for attack detection in smart homes” section. “Results and discussion” section discusses the theoretical proof for evaluating the proposed system, and finally, “Conclusion” section concludes the paper.

## State-of-the-art

In this section, the relevant works corresponding to a domain of threat modelling known and unknown threats in the smart environments are retrospected. The literature manifests that detecting cyber-threats in IoT mainly focuses on known threats. For example, Pajouh et al.^16^ employed Linear Discriminant Analysis and Principal Component Analysis for dimensionality reduction, with a two-tier classification approach comprising K-Nearest Neighbor (certainty Factor version) and Naïve Bayes to find the system state of being under threat or not. In another approach, Gao et al.^17^ hired an adaptative version of Principal Component Analysis and the Incremental Learning, i.e., an extreme learning machine to identify suspicious behaviors in smart set-ups. Later, Kumar et al.^18^ proposed an intelligent decentralized scheme for threat detection, working on fog nodes and tested on real IoT datasets. Another fog-based approach for threat detection proposed by Muder et al.^19^ used adaptive cascaded filtering and the recursive neural networks, where each network changes to different parameters/hyperparameters to expedite the detection of specific types of incursions. In continuation, Khraisat et al.^20^ proposed a hybrid approach by bringing together a decision tree (C5 classifier) and support vector machine (One-class) professing to detect both known and unknown threat variants, tested on NSL-KDD dataset. Then, Javeed et al.^24^ proposed a software-defined network-based hybrid approach with deep learning models to detect cyber threats part and parcel of smart systems. In another recent work, Al-Hawawreh et al.^21^ employed a deep learning-based pattern extractor for IoT networks with threat intelligence-based mechanisms for threat-type detection, evaluated on TON IoT and N-BAIOT. However, none of the state-of-the-artwork targeted unknown threat variants.

From the last few years, TL has emerged as a potential ground to form an invariant representation of data traffic from different domains to understand the threat insinuations of unknown types well. For example, Zhao et al.^25^ proposed a linear transformation-based TL (HeTL) model for detecting the network attacks. Still, this model requires hyper-parameters to be pre-set manually. Following this work, the authors proposed another TL model to detect unknown attacks using a cluster-based approach (CeHTL)^26^. On a similar approach, Zhang et al.^15^ proposed a domain adversarial NN-based TL approach to detect the network attacks in a smart grid, with experimentation on the created testbed. In the same line, Li et al.^22^ proposed an adaptation regularization-based TL model to detect new unseen variants of known malware. Then, Vu et al.^23^ proposed an auto-encoder-based TL approach to detect the network attacks in IoT. Later, Singla et al.^27^ used adversarial domain adaptation to develop the TL model to use the knowledge attained from one NID dataset for another domain/network with the less labeled dataset. The existing literature essentially focused either on modelling the known threats or the common vulnerabilities being part and parcel of smart devices. The authors have proposed several learning-based approaches that can classify known and unknown threats. However, some threats which do not belong to the class of the threats on which the model is being trained, remains undetected. Their work could be further improved by applying TL. The applications of TL are widely seen in areas of image identification and natural language processing, but studies using TL in threat detection are still lacking. Moreover, the applicability of the few discussed works in TL is limited due to the use of structured datasets like NSL-KDD or validating through hypotheses on different threats within the same dataset. Therefore, improving the competence of learning models for self-adaptation in diverse operating scenarios with TL for their deployment in different smart applications is the main goal of this work.

Seeing the potential of TL and the limitation of general ML-based classifiers to detect unknown threat variants, we have proposed a TL-based threat model. The proposed threat model employed the basic ML classifier with deep transductive learning. In the proposed model, the knowledge of known threats is used to detect unknown threat types, with the TON dataset comprising different attack types.

## The proposed threat modelling methodology

This section covers the threat modeling process with DFDs up to level 2.

**Figure 2:**
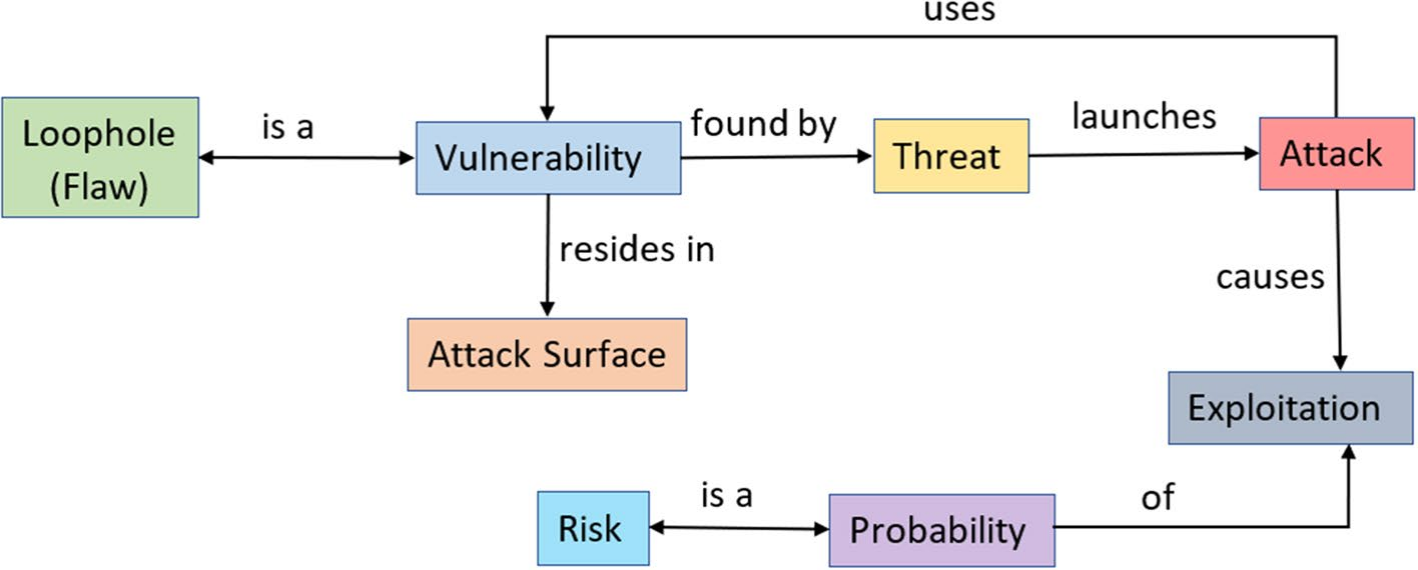
Relationship between basic terms used in Cyber-Security.

Threat Modelling is the structured process of identifying the threats in a system, communicating them to the organization’s stakeholders or end-users, and the mitigation controls to handle them. A threat can be anything that has the potential to exploit the vulnerabilities and breach the system’s security to hamper the object of interest in multiple ways. Figure 2 shows the relationship between the basic terms like vulnerability, threat, attack surface, attack, and risk in threat modelling. The different threat modelling methods like STRIDE, DREAD, VAST, etc., are widely used for identifying the threats in a system^28^. These methods are integrated into TM tools like Microsoft Threat Modeling tool^29^, ThreatModeler^30^, etc. The Microsoft TM tool designs the DFD for the system and then employs the STRIDE^31^ methodology to identify the threats at the design level. The STRIDE model covers the threats under spoofing, tampering, repudiation, information disclosure, DoS, and elevation of privileges.

On the contrary, the ThreatModeler tool designs process flow diagrams for the system under investigation and then employs the VAST^32^ methodology to identify the threats at the application/process level. The VAST model does not categorize the threats and is defined as visual, agile, and simple threat modelling. But none of these TM processes cover identifying unknown threats in their threat modelling process. The proposed threat modelling methodology works in six phases discussed as follows.

- *Find Target of Evaluation* (*ToE*): The first step while designing the security structure/model is to understand the environment your device works within and identify the use case that will be called your target of evaluation. In the proposed methodology, the smart home application and the smart devices are our ToE. In particular, we can start with the smart camera and the applications aiding the user interface as ToE.
- *Identify Assets:* After identifying your evaluation target, identify the components of the targeted device that need to be protected, which will be called the assets of a smart device. For example, in the context of the smart camera, the identified assets include log-in credentials, private keys, firmware, system configurations, network communications, event logs, and resources of the targeted device like battery, computing power, storage, and bandwidth, etc. A list of identified assets will be stored in the database. Additionally, identify the users and other entities interacting with the smart device/application.
- *Identify Threats:* Once the targeted device and assets are identified, known and unknown threats will be detected by the intelligent module based on the network traffic generated by the device and its behaviour under threat. The respective threat signal will be sent to the alert generator if the system is under threat. Also, the identified threats will be communicated to the mitigate controller, and the list will be stored in the database for further assistance.
- *Generate Alert:* Suppose the system/device is found under some known/unknown threat. In that case, the threat identification module will generate an alert to communicate to the organization’s security team or end-users about the same through email/ text message. Also, the logs of the generated threats will be stored in the database.
- *Mitigate Controller:* After the identified threats are communicated to the mitigate controller, it addresses the threats by applying the control measures from the database. Additionally, if the new security measures are created to mitigate the unknown threats, they are stored in the controller database.
- *System Validation:* The modules created for identifying known/unknown threats and those for mitigating these threats undergo a validation process before deployment in real systems by uploading on the raspberry-Pi working on the smart home gateway. The validation results are shown in performance metrics on the display unit.

**Figure 3:**
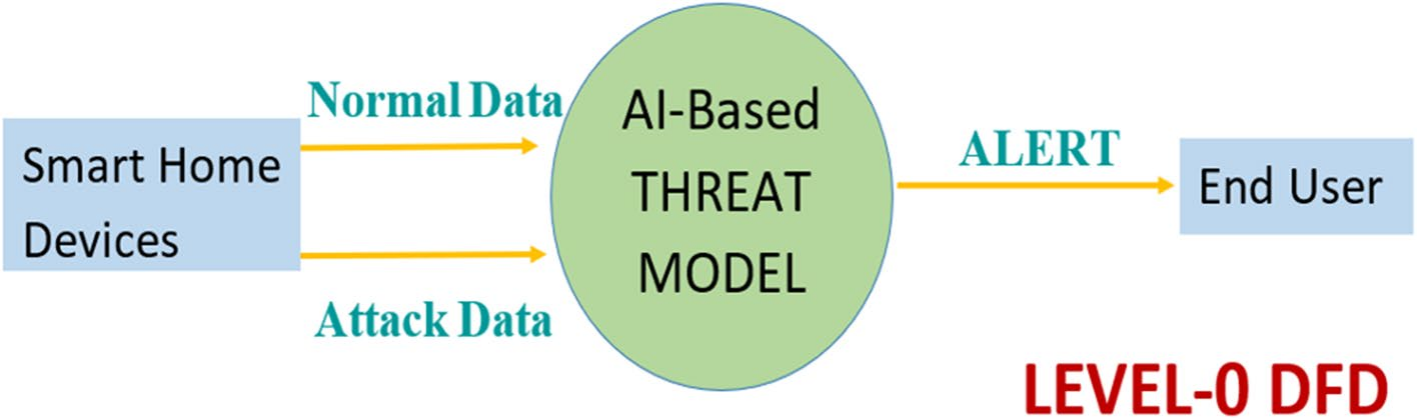
*DFD*: The level-0 DFD.

**Figure 4:**
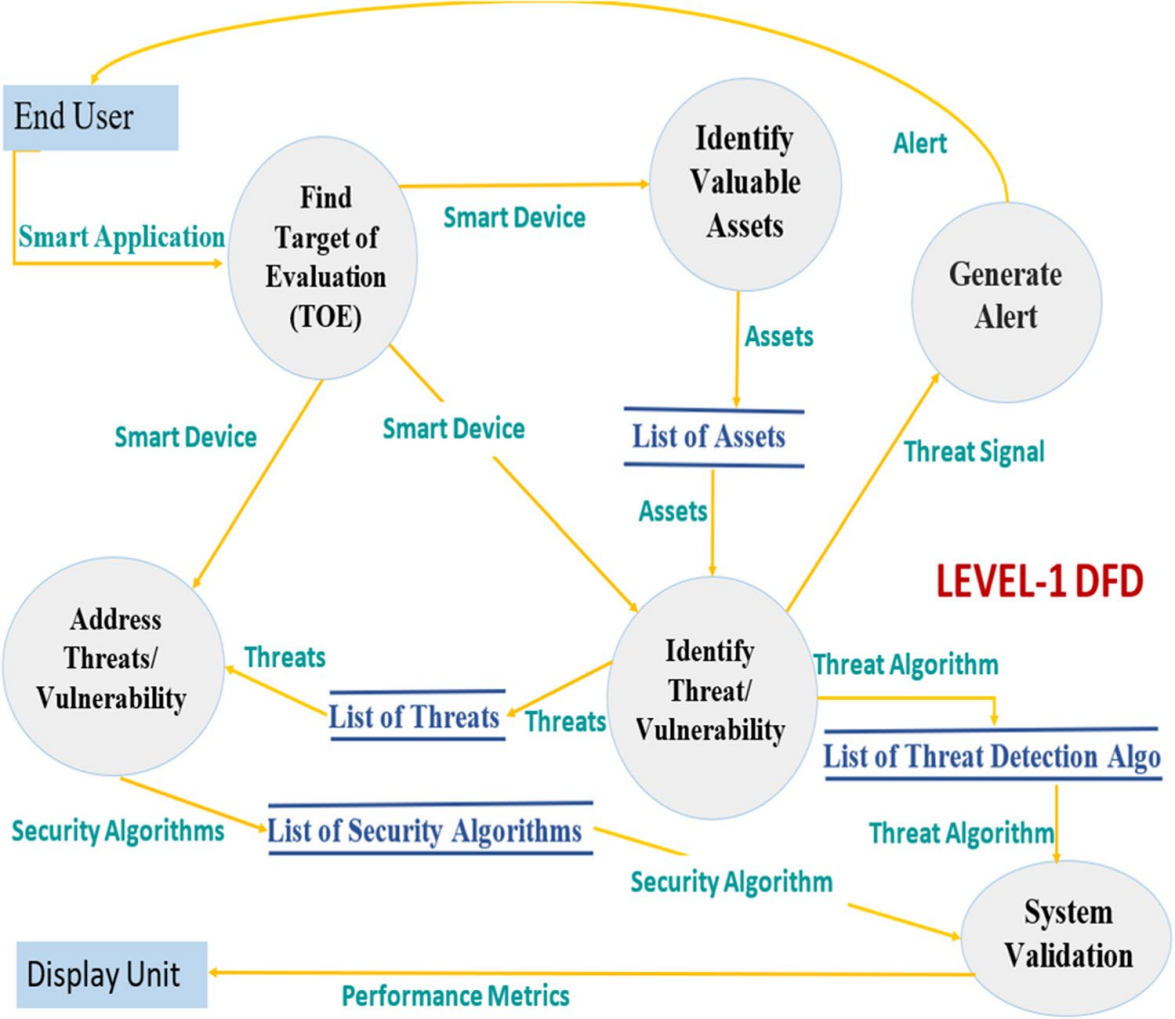
*DFD*: The level-1 DFD.

The proposed threat modelling process is also illustrated with the DFDs upto level-2. Initially, DFDs were developed for system engineers to visualize better how a system works with multiple applications in terms of moving data, applying operations on data, and storing data. In DFDs, the circle represents a process, the closed rectangle represents an entity, the rectangle with open endings represents a datastore, and the arrow represents a data flow. Figure 3 shows level-0 DFD gives us the abstract view of the entire system. It represents the entire threat model as a single process, i.e., an AI-based threat model with its relationship to external entities, i.e., smart home devices and end-users. With normal and attack data as input from the smart home devices and sending an alert as an output to the end user. Figure 4 shows level-1 DFD decomposes level-0 process into different sub-processes, which are briefly explained as the six phases of the threat modelling methodology. Thus, DFD-1 highlights the system’s main functions with processes, external entities, inputs, outputs, and data stores. At this level, the data stores are added for storing the list of threats, list of threat detection algorithms, list of alerts, and the list of threat mitigation algorithms.

**Figure 5:**
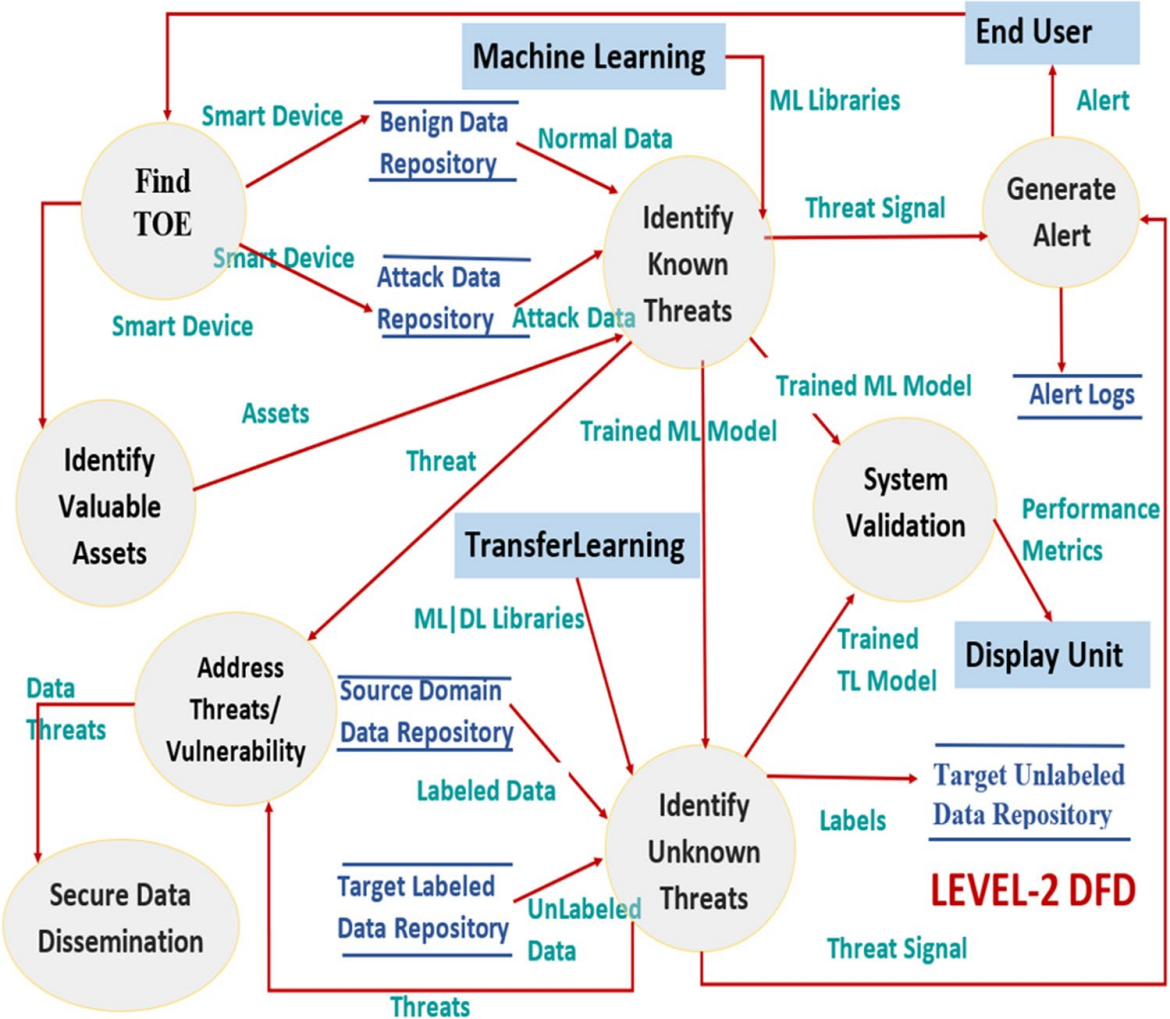
*DFD*: The level-2 DFD.

In level-2 DFD, the processes of level-1 DFD are dug one step deeper to get a clear understanding of the sub-processes further work and if they are further decomposed to the sub-modules/processes to achieve the desired functionality. At this level, the necessary details about the exact functioning of the system are recorded. Figure 5 shows the proposed threat model with level-2 DFD. The end-user selects the smart home application to check whether it is under threat or not. A specific smart home device like a smart camera or ecobee thermostat is targeted for evaluation. Then, the assets for ToE are identified to be fed to another sub-process, i.e., Identifying known threats (IKT). Also, IKT gets the data of the targeted smart device as input from the respective benign data and attack data repositories. This sub-process uses ML models to analyze the data further and generates the threat signal if the system is found under threat. These threat signals are stored in the alert logs repository, and the alert is sent to the end-user.

The identified threats are forwarded to the mitigate controller, which further addresses the threats like data integrity by applying the secure data dissemination scheme. Then, the IKT module undergoes a validation process to know the performance of the trained ML model. The trained ML model only detects the known threats with significant results on validation. The unknown threats remain undetected as the model is not familiar with the new variants of threats with different data distribution depicted by results in “SALT: the theoretical proof” section. Thus, for handling unknown threats, another TL-based learning module is added. The TL-based model takes the input as labeled data from the source domain data repository and unlabeled data from the target domain data repository. As an output, it generates the labels for the target unlabeled data and stores them in the corresponding repository. And thus sends the threat signal for the target labels which are categorized to be threats. The trained TL model also undergoes a validation process; this module will be covered in future work.

## *SALT*: transfer learning-based threat model for attack detection in smart homes

**Figure 6:**
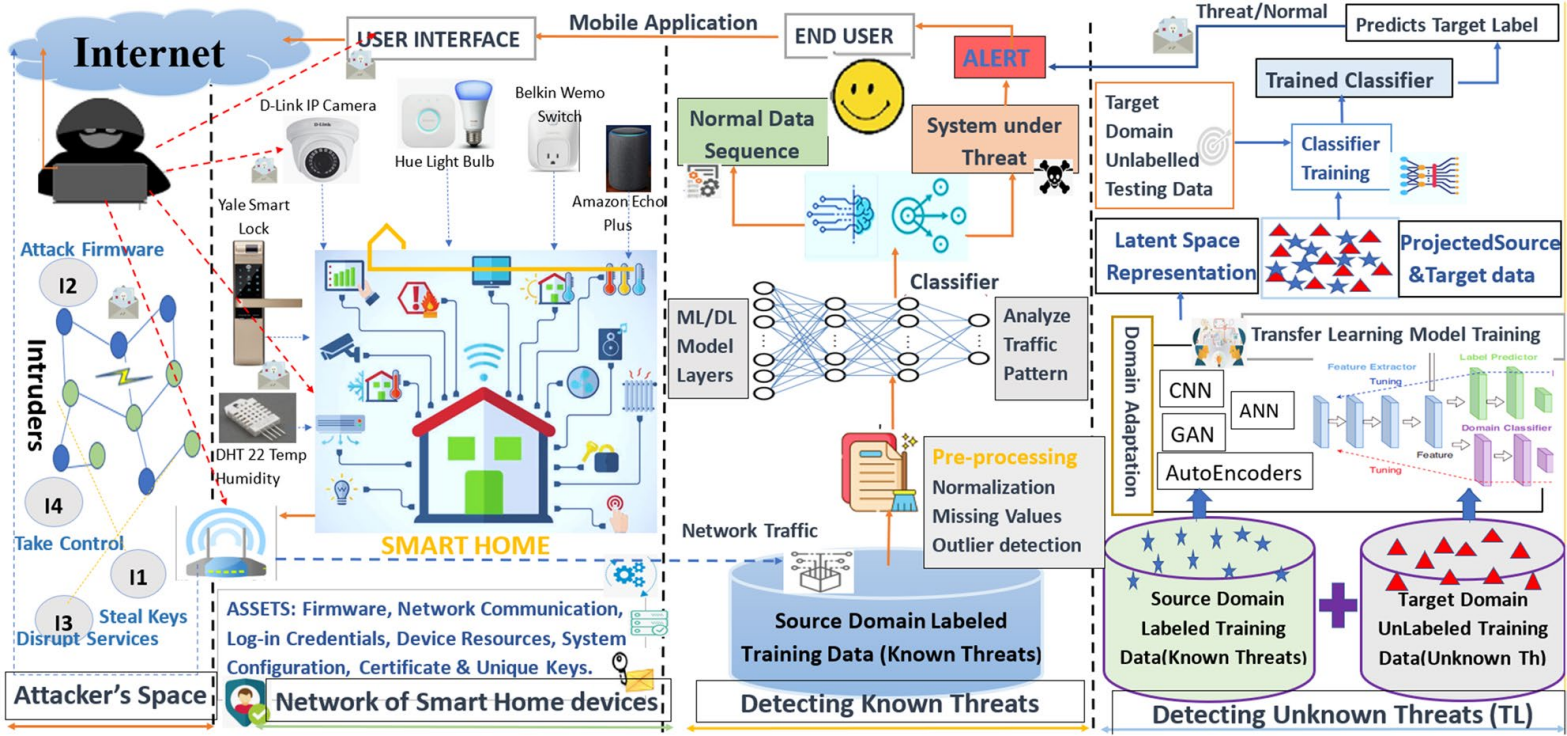
*SALT*: The Threat Model.

Figure 6 shows the threat model with learning-based robust mechanisms for threat detection in an IoT environment created in the smart home. The smart home application ($SH_m$) comprises multiple smart devices/nodes ($N_q$) connected via a network ($N_w$). Each smart device has multiple assets ($A_t$) like firmware, network communication, log-in credentials, device resources, system configuration, certificates, and unique keys that the adversaries target. The smart networks have been the victim of known and unknown threat variants rooted by the network of intruders ($I_d$) working on common attack surfaces like gateways, user interfaces, open ports, and weak authentication mechanisms. It is alarming to entrust human lives and their wealth to utilize the exploding smart technologies with poor robust security mechanisms. In the given threat model ($M_{s_y}$), the known threats ($K_m$) are detected by training the ML model with data collected from the smart home network. On the contrary, the unknown threat variants ($Z_r$) for which the machine learning classifiers ($ML_c$) underperforms, for the common classifiers like ANN and SVM, could be detected by employing domain adaptation in the TL paradigm. It is evident from the literature that unknown attack variants could be detected by leveraging the knowledge from known attack variants^33^.

Algorithm 1 presents the steps to find the known and unknown threats by applying the domain adaptation paradigm of TL. The overall time and space complexity of the given algorithm is *O*(*nLogn*) and *O*(*nd*) respectively, where *n* represents the total number of data samples and *d* represents the number of features representing each data sample in the dataset. In the given algorithm, the source domain ($D_s$) with labeled data ($L_d$) depicting known threats, whereas the target domain ($D_t$) with unlabelled data ($U_d$) depicting unknown threat variants are taken as input. The TL-based model ($TL_m$) is used to transfer the knowledge of known threats $K_m$ from the source domain, i.e., $D_s \in E_d$ to detect the unknown threats $Z_r$ in the target domain, i.e., $D_t \in E_d$, by bringing the data into the common latent space by reducing the distance between the two distributions. $L_d$ and $U_d$ are taken as input by $TL_m$ and produce $Z_i$ as output. Further, the domain invariant representation ($Z_i$) is taken as input by the learning classifier to label the target data $D_t$ as a threat or normal. Thus, TL techniques have been employed in SALT. Such schemes have the grounds to reduce the dependence on labeled data, expedite the learning speed, and strengthen the learning classifiers’ robustness against different wireless environments. The proposed threat model is elaborated in detail as follows.

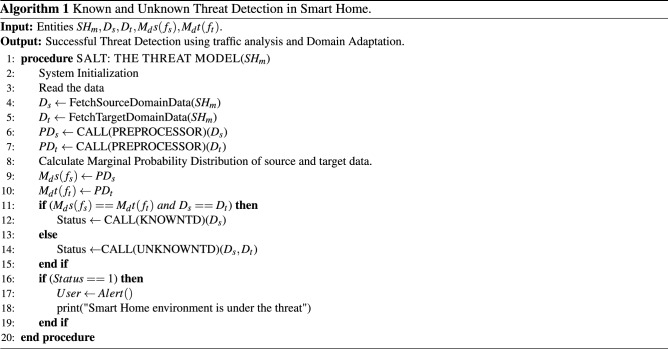

**Figure 7:**
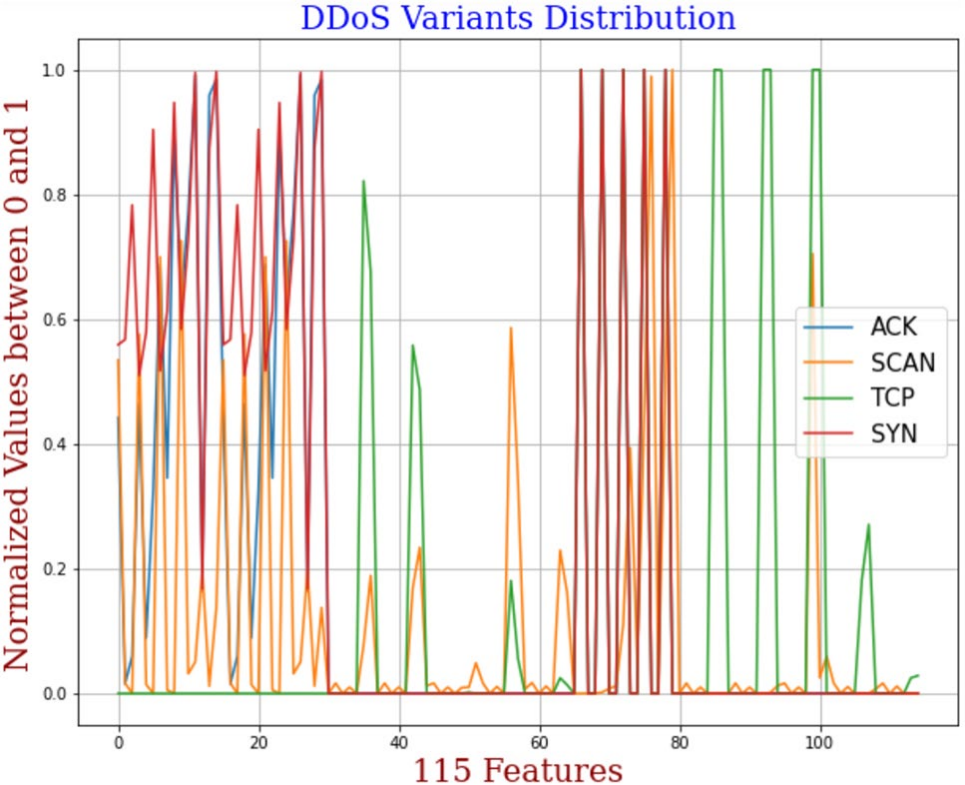
DDoS variants distribution.

A. *Attackers Space:* Smart homes are one of the reimbursements of the technological advancements in ICT with technological shifts in our personal lives. Transforming homes into smart homes with IoT and other ubiquitous technologies has widened the attack surface for the adversaries to move into the smart home remotely. This is because attackers and our smart devices are working on the same common platform, i.e., the Internet, and thus easily accessible. The attack surface of the smart home mainly includes sensors and actuators, smart devices firmware, an application installed on smartphones, the web interface, gateways, hubs, and the requisite communications within the smart home. A single intruder or chain of intruders can plan bigger attacks whose aftermaths result in bigger ransoms or denial of crucial services. Some of the threats they pose are summarized like the basic function of a smart device can be hampered by maliciously modifying the Integrated Circuit using a hardware trojan. The secret keys could even be generated by using the side channel information about the device like power dissipation, processing time, and power consumption. The clones of smart nodes are added to mislead the entire smart system and can drain the battery of sensor nodes to make them non-functional. Also, it can have an active ear over the communication links to gain the control information like node configurations, node identities, and shared network passwords. Even the communication links could be jammed, and the fraudulent packets are made the part of communication^34^.
B. *Network of Smart Home Devices:* A smart home is a housing environment equipped with several technologies to facilitate the users of interest to control home appliances and systems from anywhere. For this, smart homes communicate in real-time, whether it is about sending the sensor’s data/camera’s feeds to the user or receiving the command from the user to set the temperature at home. These intelligent services are provided by communication between the devices at home and the related environments. In the proposed threat model, the smart home is made up of devices like a smart lighting system, smart hue bulb, ecobee thermostat, Enno doorbell, smart camera, baby monitor, smart door lock, temperature/humidity sensors, and amazon echo plus. The user can access these devices through his mobile application, as they all are connected to a gateway for remote access. As per the report from Strate cast, numbers stipulate the market growth of smart homes to exceed 7 billion dollars by 2025^35^. But these devices have significant inherent vulnerabilities and are easy targets for adversaries to play with. For example, TVs and refrigerators were hacked to send spam and phishing messages. On a similar note, leading to threats like data forgery, gaining unauthorized access, and control over the smart device. Thus, the assets of the smart home environment, whether physical or information, must be handled carefully. The physical assets cover smart devices, IoT hubs, gateways, cloud servers, sensors, and actuators. The information collected by sensors, user credentials, encryption keys, certificates, installed assets, location information, etc., come under the information assets.
  In the proposed smart home threat model, there is a set of different entities{$E_a, E_n, E_w,E_i,E_t, E_k, E_z, E_m, E_f, E_d\} \in E$, where an IoT application $A_p \in \{A_1, A_2, \ldots A_a\} \in E_a$, with multiple IoT nodes $N_q \in \{N_1, N_2,\ldots N_n\} \in E_n$ is connected via a network $N_w \in E_w$. These smart home nodes $N_q$ have been the victim of known threats $K_m \in \{K_1, K_2,\ldots K_k \} \in E_k$, and unknown threats $Z_r \in \{Z_1, Z_2,\ldots Z_z\} \in E_z$. Since with time ’t’ different variants i.e., $Z_r$ of known threats $K_m$ are emerging which can have different marginal distribution $M_d \in E_m$ and feature space $F_s \in E_f$. The relationship among the aforementioned entities in the proposed threat model is represented as follows.
  1 $$\begin{aligned}&K_m ~ \xrightarrow {\alpha } ~ \sum _{i=1}^{v} A_p ~ and ~ K_m ~ \xrightarrow {\beta } ~ \sum _{j=1}^{w} N_q \end{aligned}$$
  2 $$\begin{aligned}&Z_r ~ \xrightarrow {\rho } ~ \sum _{i=1}^{v} A_p ~ and ~ Z_r ~ \xrightarrow {\sigma } ~ \sum _{j=1}^{w} N_q \end{aligned}$$
  subject to constraints
  3 $$\begin{aligned}&z \lll k ~ and ~ z \ge 0 \end{aligned}$$
  4 $$\begin{aligned}&v \le a ~ and ~ w \le n \end{aligned}$$
  5 $$\begin{aligned}&a,m,f,n,w,k \ge 1 ;d \ne 0 \end{aligned}$$
  where $\alpha$ is the association of a known threat $K_m$ with the IoT application $A_p$, and $\beta$ signifies relation with IoT nodes $N_q$. Similarly, the association of the unknown threat is shown by $\rho$ and $\sigma$. Further, the domain and the task are defined as follows. A domain *D* is defined with two terms, i.e., the feature space *F* and the marginal distribution $M_d$(f), such that their relationship is represented as follows.
  6 $$\begin{aligned} D= \{F, M_d(f)\}, ~\forall ~F = \{f_1, f_2, \ldots ,f_n\}\in F, \end{aligned}$$
  where n is the number of feature vectors.
  For different domains say $D_s$ and $D_t$, as considered in this paper, either the feature space will be different or the marginal distribution represented as follows.
  7 $$\begin{aligned}&F_s \ne F_t ~~or \end{aligned}$$
  8 $$\begin{aligned}&M_ds(f_s) \ne M_dt(f_t) \end{aligned}$$
  Given domain *D*, a task *T* is also defined with two terms, i.e., the label space L and the prediction function $P(f_i)$, which learns from the pair of feature vector and the label,i.e., {$f_i$, $l_i$} such that $f_i \in F$ and $l_i\in L$. Thus the task is represented as follows.
  9 $$\begin{aligned} T= \{L, P(f_i)\}, ~\forall ~L= \{l_1, l_2, \ldots l_m\}\in L, \end{aligned}$$
  where *m* is the number of different labels.
  For different tasks, say $T_s$ and $T_t$, either the label space will be different or the conditional distributions for the source and the target domains will be represented as follows.
  10 $$\begin{aligned} L_s \ne L_t or \end{aligned}$$
  11 $$\begin{aligned} C_ds(l_s|f_s) \ne C_dt(l_t|f_t) \end{aligned}$$
C. *Detecting known threats with machine learning:* The proposed threat model leverages ML/DL to find the status of a smart system of being under threat or not. It accepts the traces of network traffic as input from the gateway of the smart home environment that could be benign samples or attack traces of DDoS attacks like Mirai or bashlite (scanning, udp, tcp, ack, syn). The labeled source data ($U_d$), with n values, undergo data cleaning/preprocessing (outlier removal, handling missing value, encoding, and normalization) before being fed to the ML model to increase the prediction accuracy in threat detection. The procedures involved in the data cleaning process are represented as follows.
  12 $$\begin{aligned} \psi ~= ~Q_3~-~Q_1 \end{aligned}$$
  13 $$\begin{aligned} Q_1~= Median~ [1,\frac{n}{2}) \end{aligned}$$
  14 $$\begin{aligned} ~Q_3~=~Median~ \left[\frac{n}{2}~ + ~1,n \right] \end{aligned}$$
  where $\psi$ is the midspread, called the inter-quartile range, which removes the outliers from the dataset, the first quartile *Q*1 is the median of the lower half of the ordered dataset, the second quartile *Q*2, is the median value, and *Q*3 is the median of the upper half of the ordered dataset.
  15 $$\begin{aligned} Z_i~=~ \frac{f_i- max(f_i)}{max(f_i)-min(f_i)} \end{aligned}$$
  where $z_i$ is the normalized value of the feature $f_i$, which brings the numeric value of the feature of data to scale between 0 and 1. And $max(f_i)$ and $min(f_i)$ gives the maximum mad minimum value in $f_i$, given its range. Then, one hot encoding is used to generate binary values $(B_0^1)$ for the categorical features in the dataset.
  16 $$\begin{aligned} O ~ \xrightarrow {\zeta } ~B_0^1 \end{aligned}$$
  where O is the set of categorical feature values, $(B_0^1)$ is the binary output values after one-hot encoding, and $\zeta$ signifies the convert relation.
  Once the data cleaning is done, the preprocessed data will be passed to the learning algorithm (Decision tree, KNN, SVM, Random Forest, DBN, Ensemble, DNN, LSTM) for threat prediction and classification. Algorithm 2 presents the steps of preprocessing. If the system is found under threat, an alert will be generated and will be passed to the end-user through email or text message. Algorithm 3 presents the procedure to find the known threats with a learning classifier. Although, subsequent studies with ensemble learning and neural networks have performed well with good accuracy. However, these prevailing approaches result in a high false alarm rate and work under the default assumption that both training and predicting datasets follow the same probability distribution with the same threat variants in both datasets. But, this assumption fails to hold, thus degrading the accuracy of the traditional machine learning approaches. Figure 7 depicts different variants of DDoS attacks that have a different distribution, which when falls under the different datasets like training and testing results in poor performance of ML models. The proposed TL-based threat model allows the trained ML/DL classifiers to retain robust performance even against unknown threats.
D. *Detecting unknown threats with Transfer Learning:* SALT leverages transductive TL to expand the existing knowledge attained during the learning process of known threats, against unknown threats in the smart home, as a binary classification problem, classifying the events as benign or threat. This phase of the threat model follows the pipeline, executed in four steps. (i) Firstly, collect the labeled source data, i.e., known threats, and the unlabeled target data, i.e., unknown threats from their respective repositories. Then, this data undergoes various phases of pre-processing before moving further as an input to learning models; (ii) The models like GAN, ANN, Autoencoders, and CNN are trained in a manner to accomplish the domain adaptation over both the domains, to provide a new feature representation of the source and target data. For this, certain loss functions like maximum mean discrepancy are calculated, which are backpropagated to adjust the weights of neural networks till the objective function is minimized; (iii) Then, the labeled data with the new feature representation is used to train the classifiers; (iv) After the model is trained with new feature space, then map the new unlabeled target data to the new feature space, and the trained model will predict their class labels. Algorithm 4 explains the detailed procedure of detecting unknown threats with TL. This phase of the proposed threat model covers the problem of detecting unknown threats, which can be represented as follows. Given a source domain $D_s$, a source task $T_s$ with labeled traffic data $L_d$ and a related target domain $D_t$, a target task $T_t$ with unlabeled traffic data $U_d$, the transductive TL is applied to learn the target predictive function $P_t(.)$ from labeled source data and unlabeled target data to correctly predict the target labels in $D_t$. subject to constraints
  17 $$\begin{aligned}&D_s ~\ne ~D_t~ ~or~ ~M_ds(f_s) ~\ne ~M_dt(f_t) \end{aligned}$$
  18 $$\begin{aligned}&T_s ~= ~T_t ~and~ L_s~=~L_t \end{aligned}$$
  With labeled source domain traffic data $L_d$ and unlabeled target domain traffic data $U_d$, represented as follows.
  19 $$\begin{aligned} D_s~ = ~\{(f_1,l_1), (f_2,l_2), \ldots (f_n,l_n)\} \end{aligned}$$
  20 $$\begin{aligned} T_s = \{f_{n + 1}, f_{n+2}, \ldots , f_{n+m}\} \end{aligned}$$
  And the target predictive function $P_t(.)$ with least expected error on $D_t$ given by
  21 $$\begin{aligned} P_t(.) ~:~ f_t~\rightarrow ~ l_t \end{aligned}$$

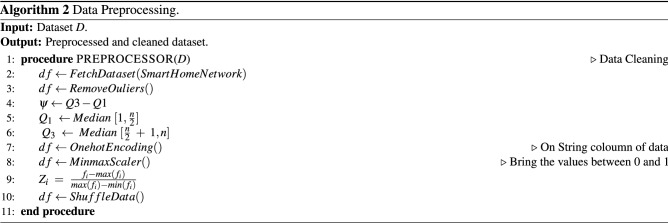

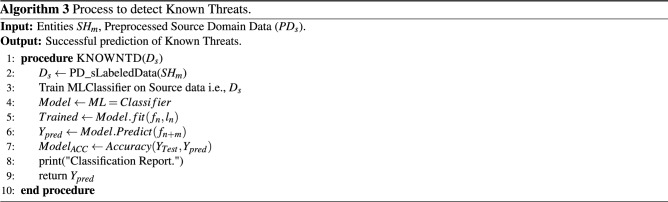

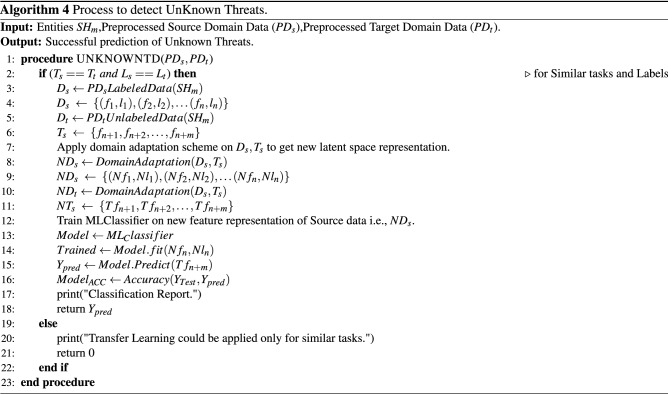







## *SALT*: the theoretical proof

*Problem statement* Smart home is the most adaptable IoT structure in the coming years. But, this transformation of homes into smart homes has widened the attack surface for the adversaries to remotely move into the smart home. Mainly, a smart home involves two different tasks one is data processing and the other is security. In this problem statement, we majorly focus on the security aspect. So, our focus in this paper is to detect both known and unknown threats in the smart home.

*Assumption* We are considering the unknown active attacks targeting the smart home. These attacks are visible/sensible to users as attackers take some action affecting system resources like services being denied and the creation of dumb files that the system administrator can sense the system is being attacked or under threat.

*Null hypothesis* $(H_0)$ An AI-assisted threat model to detect the unknown threats.

*Alternate hypothesis* $(H_1)$ An AI-assisted threat model can not detect unknown threats.

*Hypothesis testing* Let $M_1$ be the model to detect known threats, whose signatures have been stored in the database of known threats represented as follows.

22 $$\begin{aligned}&M_1 \leftarrow E_k ~ and \end{aligned}$$

23 $$\begin{aligned}&E_k \leftarrow \{K_1, K_2, \ldots K_k \} \end{aligned}$$

Taking a sample of known threats $S_k$ from the population $E_k$, such that a threat is represented by the set of ’t’ features.

24 $$\begin{aligned}&S_k \subset E_k \end{aligned}$$

25 $$\begin{aligned}&S_k \leftarrow \{F^1_1, F^1_2,F^1_3, \ldots F^1_t\} \end{aligned}$$

Let $M_2$ be the model to detect unknown threats, after transferring the knowledge of known threats from the model $M_1$ represented as follows.

26 $$\begin{aligned}&M_2 \leftarrow M_1 + \delta ~ and \end{aligned}$$

27 $$\begin{aligned}&\delta \in \{Z_1, Z_2, \ldots Z_z\} \in E_z \end{aligned}$$

Taking a sample of unknown threats $U_k$ from the population $E_z$, such that a threat is represented by the set of ’t’ features.

28 $$\begin{aligned}&U_k \in E_z ~and~ U_k \notin E_k \end{aligned}$$

29 $$\begin{aligned}&U_k \leftarrow \{F^2_1, F^2_2,F^2_3 \ldots F^2_t\} \end{aligned}$$

**Figure 8:**
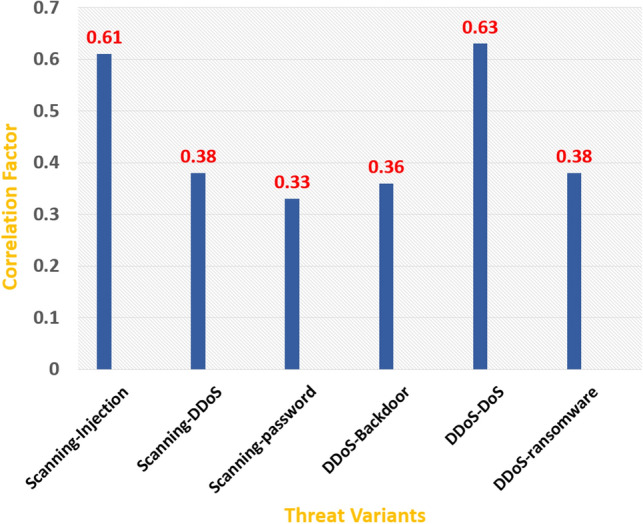
Correlation between Unknown Threats.

Then, we apply the Pearson correlation coefficient, to find the correlation of new threat $U_k$ with existing threats $E_k$.

30 $$\begin{aligned} r(F^1_i, F^2_i) = \frac{ N\sum {F^1_i F^2_i}-\left( \sum {^1_i}\sum {F^2_i}\right) }{ \sqrt{ \left[ N \sum {x^2}-\left( \sum {x}\right) ^2 \right] \left[ N \sum {y^2}-\left( \sum {y}\right) ^2 \right] } } \end{aligned}$$

where *N* is the number of sample rows and $x \in F^1_i, y \in F^2_i$

31 $$\begin{aligned} r_s(S_k, U_k)= & {} \sum _{i=1}^{t} r(F^1_i, F^2_i) \end{aligned}$$

32 $$\begin{aligned} r^{'} _s(S_k, U_k)= & {} Maxminscaler(r_s) \end{aligned}$$

where $r^{'} _s(S_k, U_k)$ is the normalized value of the correlation coefficient. The final state i.e., $R(SH_m)$ of the system being under the threat or not is given as follows.

33 $$\begin{aligned} R(SH_m) = \left\{ \begin{array}{lr} S_k, &{} \text {for } r^{'} _s\ge \theta \\ U_k, &{} \text {for } r^{'} _s < \theta \end{array}\right\} \end{aligned}$$

where the threshold, i.e., $\theta$, is decided by finding the correlation coefficient between samples of known and unknown threats. If the correlation coefficient, i.e., $r^{'} _s(S_k, U_k)$ is higher than $\theta$, then the system is found to be under the known threat, and for values, $r^{'} _s(S_k, U_k)$ lesser than $\theta$, the system is found to be under the unknown threat. For deciding the threshold, we have considered the TON-IoT dataset. In this dataset^36^, the subset containing the network traffic of the IoT network of devices like fridges, thermostats, garage doors, GPS trackers, Modbus, weather, and motion light is used in the experimental analysis. The experiment is run for critical analysis for six different attack categories, including backdoor, the man in the middle attack, DDoS, DoS, injection, and password. Different samples are taken with relevant features to find the correlation between the known threats from each category. Then, the Pearson correlation matrix is computed using the corrcoef function of the NumPy library in Python. From the results, it is observed that for known threats, the correlation factor is found to be greater than $65\%$. And for unknown threats, the correlation factor for samples from different threat categories is observed to be less than $63\%$, which is also depicted in Fig. 8. Thus, from the experimental analysis, the threshold value is decided as 0.63.

## Results and discussion

**Figure 9:**
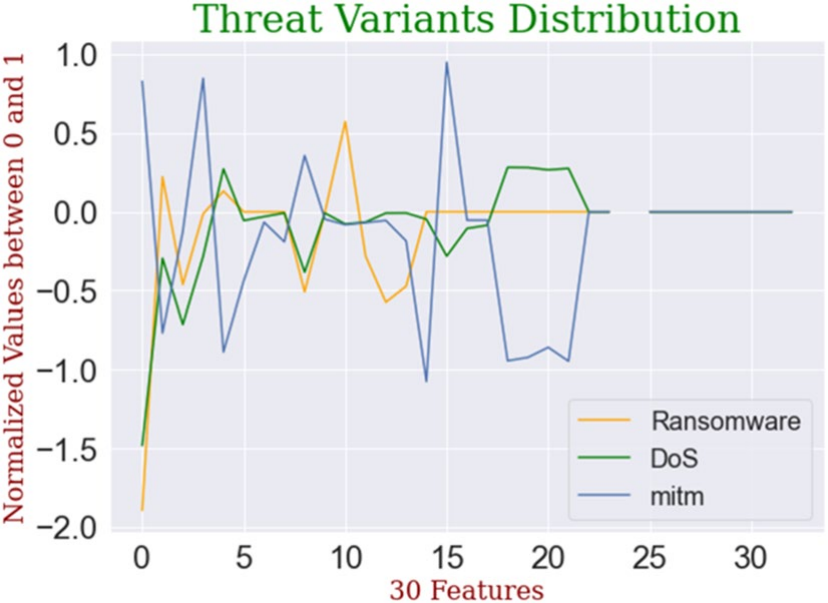
Distribution of different threats.

In this section, we evaluated the performance of general ML-based solutions to detect known and unknown threat variants. We addressed the following question: Does an ML-based classifier work well in detecting threats with unknown/different distributions? We utilized a benchmark IoT dataset-the ToN-IoT dataset, collected over distributed data sources. We carried out eight experiments to check the performance over unknown threat variants. To evaluate the performance of general ML-classifiers, we divided the network dataset of the ToN-IoT dataset into eight subsets, as presented in Table 1 for the first testing scenario. The ToN-IoT dataset consists of nine attack families, including backdoor, injection, ransomware, DoS, DDoS, password, scanning, cross-site scripting, and man-in-the-middle attack. Figure 9 shows the differences in the distribution of individual threats represented by the normalized vector of corresponding features. This dataset has the telemetry data collected from IoT nodes, with different operating System logs and network data obtained from a practical depiction of a medium-scale network at the UNSW Canberra Cyber Range and IoT labs (Australia). This is a publicly accessible dataset available at the ToN-IoT repository. These datasets were collected from the three-tier (edge, fog, and cloud) testbed comprising different IoT sensors like weather, Modbus, etc., designed to cover traces of network, process, memory, and hard disk in Windows and Linux-based IoT systems.

**Table 1:** Description of IoT datasets.

Dataset	Attacks	Size of dataset
ToN-IoT 1	dos, ddos	60,000
ToN-IoT 2	Backdoor	20,000
ToN-IoT 3	Injection	20,000
ToN-IoT 4	Ransomware	20,000
ToN-IoT 5	Scanning	20,000
ToN-IoT 6	Cross-site scripting	20,000
ToN-IoT 7	Man-in-the-middle	1043
ToN-IoT 8	Password	20,000

To analyze the behaviour of multiple classifiers on different threat distributions between the source threat domain and target unseen threat variants, we have used the network traces of the ToN-IoT dataset. Each data sample in the network dataset has 44 features covering activities such as HTTP, violation, SSL, DNS, statistical, and connection, presented in Table 2. Before training the classifier, data undergoes various preprocessing phases to remove outliers, handle missing values, and normalize the data. Then the ML model is trained on the dataset with normal data samples and two attack types and tested on the dataset with different attack types. To follow the pattern, eight experiments are conducted. For example, the classifier is trained on the dataset IoT 1 with 20,000 normal samples and 20,000 samples for each category of DoS and DDoS attacks for the first testing scenario. In the first scenario, the learning model is trained on two threats i.e., dos and ddos, and tested for the remaining seven attack categories on all datasets from IoT 2 to IoT 8, comprising different attack categories of backdoor, injection, ransomware, scanning, cross-site scripting, man-in-the-middle attack, and password, with around 20,000 samples in each category. The 30 relevant features are taken in the final dataset, presented in Table 3.

**Table 2:** Features of TON datasets.

Feature	Service profile	Feature	Service profile
Ts (timestamp)	Connection activity	Ssl_version	SSL activity
Src-ip	Connection activity	Ssl_cipher	SSL activity
Src-port	Connection activity	Ssl_resumed	SSL activity
Dst-ip	Connection activity	Ssl_established	SSL activity
Dst-port	Connection activity	Ssl_subject	SSL activity
Proto	Connection activity	Ssl_issuer	SSL activity
Service	Connection activity	Weird_name	Violation activity
Duration	Connection activity	Weird_addl	Violation activity
Src-bytes	Connection activity	Weird_notice	Violation activity
Dst-bytes	Connection activity	http_trans_depth	HTTP activity
Conn-state	Connection activity	http_method	HTTP activity
Missed-bytes	Connection activity	http_uri	HTTP activity
src_pkt	Statistical activity	http_version	HTTP activity
src_ip_bytes	Statistical activity	http_request_body_len	HTTP activity
dst_pkts	Statistical activity	http_response_body_len	HTTP activity
dst_ip_bytes	Statistical activity	http_status_code	HTTP activity
Dns-query	DNS activity	http_user_agent	HTTP activity
dns_qclass	DNS activity	http_orig_mime_types	HTTP activity
dns_qtype	DNS activity	http_resp_mime_types	HTTP activity
dns_rcode	DNS activity	label	Data labelling
dns_AA	DNS activity	type	Data labelling
dns_RD	DNS activity	dns_rejected	DNS activity
dns_RA	DNS activity

**Table 3:** Selected features of TON dataset for training.

Feature	Feature
Ts (timestamp)	Ssl_version
Conn-state	Ssl_cipher
Src-port	Dst-port
Dst-bytes	Src-bytes
Missed-bytes	http_uri
src_pkt	dns_RD
src_ip_bytes	http_request_body_len
dst_pkts	http_response_body_len
dst_ip_bytes	http_status_code
dns_RA	http_user_agent
dns_qclass	dns_rejected
dns_qtype	http_resp_mime_types
dns_rcode	proto
label	dns_AA
Ssl_established	

The authors have divided the network dataset of the ToN-IoT dataset into nine subsets comprising 1 normal subset and 8 different attack categories of dos, ddos, backdoor, injection, ransomware, scanning, cross-site scripting, man-in-the-middle attack, and password, with around 20,000 samples in each category, except man-in-the-middle attack with 1043 samples. The threats for which the model is trained for are categorized under known threats. The threats for which the model is not trained for becomes unknown to the model. The experiments are conducted for three different scenarios. In the first scenario, the learning model is trained on two threats i.e., dos and ddos, and tested for the remaining seven attack categories. For the first scenario, in terms of known and unknown threats, the training and testing ratio is 2:7. In the second scenario, the learning model is trained on four threats i.e., dos, ddos, mitm, backdoor, and tested for the remaining five attack categories. For the second scenario, the training and testing ratio is 4:5. In the third scenario, the learning model is trained on six threats i.e., dos, ddos, mitm, backdoor, password, injection, and tested for the remaining three attack categories. For the third scenario, the training and testing ratio is 6:3.

To evaluate the performance of basic ML classifiers like Artificial Neural Networks (ANN), Support Vector Machine(SVM), etc., over both the domains of known and unknown threats, we use confusion matrix and several other performance criterion like accuracy, precision, recall, and f1-score.

**Figure 10:**
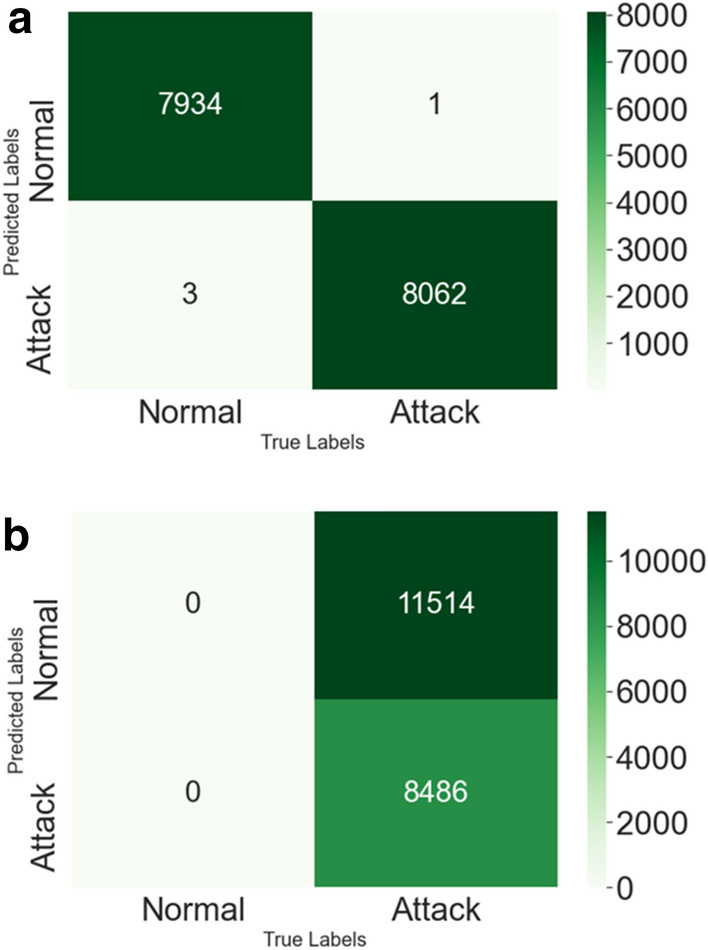
Confusion matrices for known and unknown Threats.

**Figure 11:**
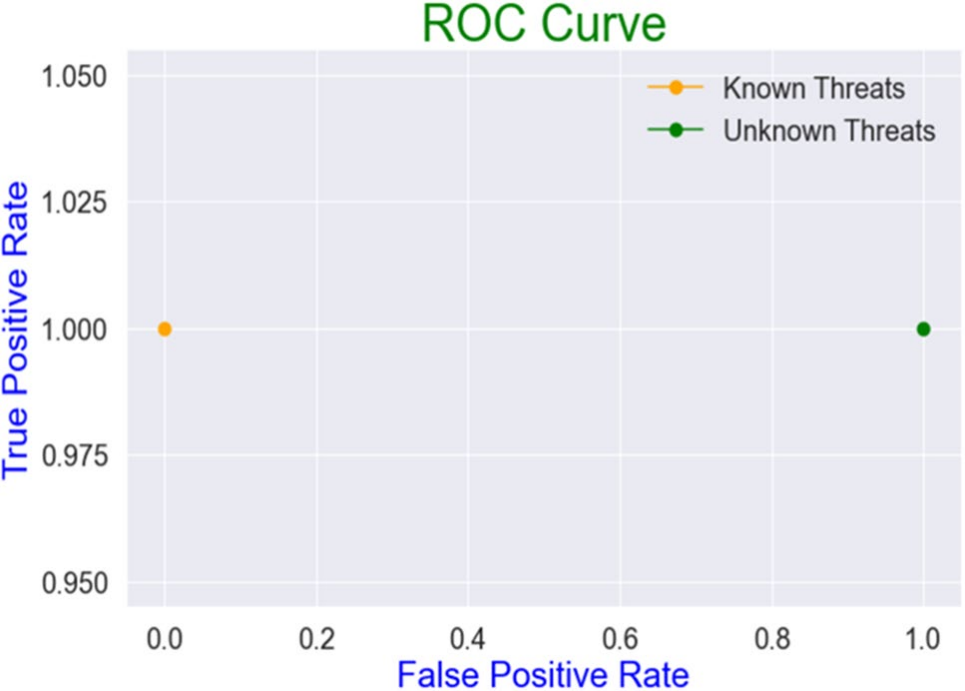
RoC Curve for known and unknown Threats.

Achieving 100% precision in all experiments in the first scenario for detecting known and unseen threat variants reflects that the classifier correctly predicts all the non-malicious/normal data samples. This indicates that the model does not have any false positives. On the contrary, lower f1-scores falling in the range of 56 to 71% show the model’s inability to classify the unseen malicious data samples correctly. Thus, extensive experiments on the network dataset of Ton-IoT validate that learning classifiers (SVM) can achieve over 100% of f1-score and recall for detecting known threats. However, it significantly underperforms for the first testing scenario, with a 56.6% of f1-score and 39.4% of accuracy for detecting unseen threat variants (cross-site scripting), presented in Table 4. Figures 10, 11 shows the roc curve for known and unseen threats. The orange dot depicts the curve for known threats, indicating zero false positives and a 100% true positive rate. The green dot depicts the curve for unseen threat variants, indicating the high false-positive rate of 100%, though with a 100% true positive rate. Although, the model also underperforms for unknown threats in the other testing scenarios, with the accuracy dropping to 41.95 in the second scenario and 61.4 in the third scenario. However, it is observed that the model outperforms for even the unknown threats in the third scenario, when the training and testing ratio is 6:3. Table 5 shows the performance of the model under the three different scenarios.

**Table 4:** ML Classifier performance on unknown threats.

Dataset	Accuracy	Precision	Recall	F1-score
IoT-1	100	100	100	100
IoT-2	51	100	51	67.5
IoT-3	46.2	100	46.2	63.2
IoT-4	40.4	100	40.4	57.5
IoT-5	42.4	100	42.4	59.5
IoT-6	39.4	100	39.4	56.6
IoT-7	55	100	55	71.4
IoT-8	49.2	100	49.2	65.9

**Table 5:** Performance on three different testing scenarios.

Dataset	T-1 (2 known:7 unknown), %	T-2 (4 known:5 unknown)	T-3 (6 known:3 unknown)
TON IoT-4	40.4	43.37	55.02
TON IoT-5	42.4	48.36	57.92
TON IoT-6	39.4	41.95	61.4

**Figure 12:**
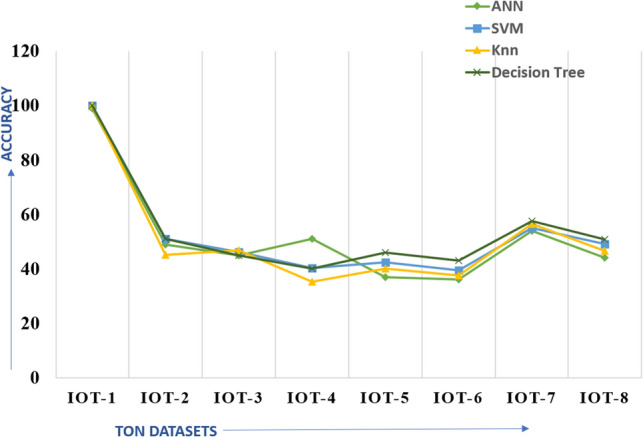
Performance on different threats.

Figure 10a shows the confusion matrix for known threat detection. Out of the normal data points, all are correctly classified as normal except one data sample classified as anomalous. Out of the anomalous data, 8062 are correctly identified as malicious, whereas 3 are classified as normal. Figure 10b shows the confusion matrix for unknown threat detection where 11,514 attack data points are wrongly classified as normal. Figure 12 shows the performance of common ML classifiers, namely, Support Vector Machine(SVM), ANN, decision tree, and k nearest neighbor (Knn), over different threat variants. It is observed that these classifiers perform well when tested on data (ToN IoT-1) having the same distribution as the training data, i.e., known threats. However, for the different distribution, i.e., unknown threats (ToN IoT-2, 3, 4, 5, 6, 7, 8), the accuracy drops from 98 to around 39%. Hence, the basic ML models underperform for threats with different distribution in comparison to the distribution of the training set.

## Conclusion

The smart home, empowered with declining costs in distributed computing technology, communications, and sensors, connects users and utilities, providing remote control and management. However, the expedited growth of interconnections among billions of smart nodes brings cyber-threats to homes with disastrous influence. Therefore, it is important to assess and mitigate these threats to avail benefits of smart services. Motivated by the discussion mentioned above, in this work, we presented a threat modeling methodology using DFDs upto level-2 depicting the process of handling known and unknown threats. Then, we have given an intelligent threat model based on a TL scheme with basic ML classifiers for known and unknown threat variants by reducing the differences in their distribution. Finally, the theoretical proof with the threshold decidability factor is given. In addition, the proposal demonstrated three distinctive testing scenarios in which the model outperform for the third scenario with training testing ratio of known unknown threats as 6:3. However, the overall results show that the traditional schemes underperform for unknown threats with different distribution with accuracy dropping to 39% and high false-positive rates for these variants.

In the future, the proposed threat model will be implemented, and domain adaptation will be employed to reduce the distance between data of different distributions for unknown threat detection. This work is confined to single smart home environment, further this work could be extended to inter-domain. Also, we can analyse the behaviour of the model by working upon the imbalanced dataset. The proposed threat model could be extended for mitigation and real-time implementation in heterogeneous scenarios.

## Data Availability

There is no data available to carry out this research work so data availability is not applicable for this manuscript.
